# Runge–Kutta Numerical Method Followed by Richardson’s Extrapolation for Efficient Ion Rejection Reassessment of a Novel Defect-Free Synthesized Nanofiltration Membrane

**DOI:** 10.3390/membranes11020130

**Published:** 2021-02-14

**Authors:** Chabi Noël Worou, Jing Kang, Jimin Shen, Pengwei Yan, Weiqiang Wang, Yingxu Gong, Zhonglin Chen

**Affiliations:** State Key Laboratory of Urban Water Resource and Environment, School of Environment, Harbin Institute of Technology, Harbin 150090, China; jingkanghit@163.com (J.K.); shenjimin@hit.edu.cn (J.S.); yanpengwei@stu.hit.edu.cn (P.Y.); wangweiqiang@stu.hit.edu.cn (W.W.); gyx667@126.com (Y.G.)

**Keywords:** zirconium nanoparticles, soft computing, salt rejection, nanofiltration membrane, Runge–Kutta numerical method, Richardson’s extrapolation

## Abstract

A defect-free, loose, and strong layer consisting of zirconium (Zr) nanoparticles (NPs) has been successfully established on a polyacrylonitrile (PAN) ultrafiltration substrate by an in-situ formation process. The resulting organic–inorganic nanofiltration (NF) membrane, NF-PANZr, has been accurately characterized not only with regard to its properties but also its structure by the atomic force microscopy, field emission scanning electron microscopy, and energy dispersive spectroscopy. A sophisticated computing model consisting of the Runge–Kutta method followed by Richardson extrapolation was applied in this investigation to solve the extended Nernst–Planck equations, which govern the solute particles’ transport across the active layer of NF-PANZr. A smart, adaptive step-size routine is chosen for this simple and robust method, also known as RK4 (fourth-order Runge–Kutta). The NF-PANZr membrane was less performant toward monovalent ions, and its rejection rate for multivalent ions reached 99.3%. The water flux of the NF-PANZr membrane was as high as 58 L · m^−2^ · h^−1^. Richardson’s extrapolation was then used to get a better approximation of Cl^−^ and Mg^2+^ rejection, the relative errors were, respectively, 0.09% and 0.01% for Cl^−^ and Mg^2+^. While waiting for the rise and expansion of machine learning in the prediction of rejection performance, we strongly recommend the development of better NF models and further validation of existing ones.

## 1. Introduction

With an ever-growing population and an increase in their standard of living and needs, as well as the expansion of industrial and agricultural activities, there is still an increasing demand for good quality water around the world. Moreover, worldwide, water scarcity is recognized as not only a present but also a future threat to human activities. To meet this increase in demand, with no loss being tolerable, a water treatment plant, in all its aspects and all its forms, needs to be optimized and sophisticated. One of the major technological challenges nowadays is the development of sustainable processes for water desalination, water reuse, wastewater treatment, and recovery of valuable chemicals from water [1,2,3]. Among membrane technologies, the pressure-driven process was reported to be effective for the separation of multivalent ions such as ${\mathrm{Mg}}^{2+},{\;\mathrm{Ca}}^{2+},{\;\mathrm{SO}}_{4}^{2-}$, etc. [3,4].

A nanofiltration (NF) membrane is a pressure-driven process exhibiting properties that lie betwixt ultrafiltration (UF) and reverse osmosis (RO) [5]. NF membranes have proven their effectiveness in several sectors such as water reclamation [6] and dye separation [7], heavy metals [8,9], pesticides [10], viruses and bacteria [11,12], beverages [13], natural organic matter [14], food [15], dairy processing [16], hardness removing [17], taste and odors [18], and even water softening [19]. Since there are several applications for NF membranes, there is, therefore, a compelling need to understand its separation behavior and how to improve the solute particles’ transport mechanisms through its active selective layer, especially in water desalination. The NF process is reported to be extremely complex and dependent on both interfacial and micro-hydrodynamic events that occur at its surface, even within its nanopores. Donnan, steric, transport, and dielectric effects, combined together, determine the NF membrane’s removal performance [20].

At the start, the dielectric exclusion phenomena were not understood very well with two competing hypotheses that tried obscurely to explain the exact nature of interaction that takes place. The first hypothesis was called the “image forces” phenomenon [21], while the second was called the “solvation energy barrier” mechanism [22]. These two exclusion phenomena have already been investigated in detail [23]. The selectivity of solute particles and the permeate flux are the two main factors that determine the membrane performance. Recent investigations demonstrated that the membrane must be highly chemical resistant, thermally stable, and loose to exhibit good separation results. NF membranes can be either symmetric or asymmetric in structure, homogenous or heterogeneous, and neutral or positively or negatively charged. The NF membrane separation process is not only faster but also more efficient and cost-effective than conventional separation techniques. The separation process using NF membranes has the following advantages: (i) energy consumption is low, (ii) separation can be performed under mild conditions, (iii) no additives are required, (iv) separation occurs continuously, (v) it is possible to combine NF process with other separation processes, (vi) the separation process can be up-scaled easily, and (vii) NF membrane properties can be adjusted.

In the present work, an organic–inorganic NF membrane has been synthesized to conciliate the tremendous advantages of both organic and inorganic membranes. A polyacrylonitrile (PAN) ultrafiltration (UF) membrane has been used as a substrate, and zirconia (ZrO_2_) nanoparticles (NPs) have been chosen for deposition under the in situ formation process. Dopamine hydrochloric/sodium bicarbonate buffers are co-deposited on the PAN platform to induce the further growth of dioxide zirconium (ZrO_2_) NPs on the membrane surface. The resultant organic–inorganic NF membrane, NF-PANZr, has been depicted as to its structure by various tools including atomic force microscopy (AFM), field emission scanning electron microscopy (FESEM), and energy dispersive spectrometry (EDS).

NF models based on the extended Nernst–Planck equations that govern the transport of solute particles through the NF membrane active layer have been imagined so far. A good model should include more of the complex phenomena that govern separation mechanisms to improve not only the physical robustness but also the relevance of the description of the process. Runge–Kutta method, a reasonably simple and robust method [24], has been used for ion rejection reassessment followed by Richardson’s extrapolation to get a better approximation of solute particle rejection.

## 2. Mathematical Modeling

In numerical analysis, the Runge–Kutta methods include both implicit and explicit iterative methods. Runge–Kutta methods are based on the reputed routine called the Euler method, used for approximate solutions of ordinary differential equations. Also known as RK4 (fourth-order Runge–Kutta), this method is reasonably simple and robust and is highly recommended for all differential equations, provided that a smart, adaptive step-size routine is chosen [24].

### 2.1. Model Assumptions

(i) Boundary conditions:
  It is assumed that Equations (5) and (9) can be solved over the following conditions:
  At x = 0, $\to \;C_{i}=C_{i,f}$
  At x = Δx, $\to \;C_{i}=C_{i,p}$
(ii) The solution understudy is ideal.
(iii) Each solute particle is subjected to an extended Nernst–Planck equation and could therefore be transportable.
(iv) The effective charge density of NF-PANZr membranes does not change from one point to another on its surface.
(v) The layer thickness of nanoparticles is assumed to be negligible toward the platform thickness.
(vi) The NF membrane consists of an identical bundle of straight cylindrical pores, with each pore displaying a uniform depth and radius $r_{p}\ll \Delta x$.
(vii) The electric potentials inside the membrane and the ${\mathrm{Na}}_{2}{\mathrm{SO}}_{4},{\mathrm{MgSO}}_{4},\;\mathrm{NaCl},{\mathrm{CaCl}}_{2},{\;\mathrm{and}\;\mathrm{MgCl}}_{2}$ solutions are all defined in terms of averaged quantities.
(viii) The Donnan equilibrium is applied at both the interface of feed solution—membrane and the interface of membrane—permeate solution.

### 2.2. Focus on Model Equations

Runge–Kutta (RK) techniques were first introduced at the beginning of the 19th century by C. Runge and M. W. Kutta. Then, shortly after, this method took a major role in the study of iterative methods based on explicit, partial, and implicit methods that were applied to solve the ordinary differential equations (ODEs) using a temporal discretization.

Through his famous book, C. Runge had the ingenious idea to develop and make more accurate Euler’s approximation method by proposing a scheme that could offer very high precision. Runge’s work was first developed by Heun around 1900 and then by Kutta in 1901. The base of this scheme is expressed as follows:

(1) $$y_{\left(f+1\right)}-y_{f}=\sum_{i=1}^{m}w_{i}k_{i}$$

$y_{\left(f+1\right)}-y_{f}$ is difference between the values of *y* at $t_{\left(n\right)}$ to $t_{\left(n+1\right)}$, $w_{i}$ are constants, and $k_{i}$ are given by

(2) $$k_{i}=hf\left(t_{n}+c_{i}h;\;y_{n}+h\sum_{j=1}^{i-1}a_{ij}k_{j}\right)$$

This is the consistency relation of the Runge–Kutta (RK) method, $\sum_{j=0}^{s}a_{ij}=c_{i}$ and $\sum_{j=0}^{s}b_{j}=1$.

The main equation to solve, which governs the solute particles’ transport across the active NF membrane active layer, is known as the extended Nernst–Planck equation and is given as

(3) $$j_{i}=K_{i,c}c_{i}J_{v}-D_{i,p}\frac{dc_{i}}{dx}-\frac{z_{i}c_{i}D_{i,p}}{RT}F\frac{d\Psi }{dx}$$

$j_{i}$ is the flux of ion [i] related to the membrane area $\left(\mathrm{mol}.m^{-2}.s^{-1}\right);\;z_{i}$, ion [i] valence; Ψ, the electrical potential within the pore, V; $D_{i,p}$ is the pore diffusion coefficient $\left(m^{2}\cdot s^{-1}\right)$, R is universal gas constant $\left(8.314\;J\cdot {\mathrm{mol}}^{-1}\cdot K^{-1}\right)$, $c_{i}$, the concentration of ion [i] within the pore $\left(\mathrm{mol}\cdot m^{-3}\right)$, dimensionless; $K_{i,c}$, hindrance factor for convection for ion [i]; $J_{v}$, volume flux related to the membrane area $\left(m^{3}\cdot m^{2}\cdot s^{-1}\right)$; T, absolute temperature K; F, Faraday constant $\left(96,487\;C\cdot {\mathrm{mol}}^{-1}\right)$.

The transport of ions through the membrane can be achieved by applying the defined boundary conditions. It is easier to assess solute particle rejection by writing the Nernst–Planck equation as concentration and potential gradients. For the concentration gradient determination, the relation between the ion flux and its concentration is depicted as

(4) $$j_{i}=C_{i,p}.J_{v}$$

where $C_{i,p}$ is the ion [i] concentration in the permeate $\left(\mathrm{mol}\cdot m^{-3}\right)$. By substituting Equation (2) into Equation (1) and rewriting, the concentration gradient is given as

(5) $$\frac{dc_{i}}{dx}=\frac{J_{v}}{D_{i,p}}\left(K_{i,c}c_{i}-C_{i,p}\right)-\frac{z_{i}c_{i}}{RT}F\frac{d\Psi }{dx}$$

Several conditions were involved in the potential gradient obtention. The electro-neutrality within the pore, the permeate solution, and feed solution are governed respectively by Equations (6)–(8):

(6) $$\sum_{i=1}^{n}z_{i}c_{i}=-X_{d}$$

(7) $$\sum_{i=1}^{n}z_{i}C_{i,p}=0$$

(8) $$\sum_{i=1}^{n}z_{i}C_{i}=0$$

where $X_{d}$ is the effective charge density of the membrane (in $\mathrm{mol}\cdot m^{-3}$).

The potential gradient is obtained taking into account the above-defined conditions in Equations (6)–(8) for the concentration gradient depicted in Equation (5). The potential gradient is then obtained by

(9) $$\frac{d\Psi }{dx}=\frac{\sum_{i=1}^{n}\frac{z_{i}J_{v}}{D_{i,p}}\left(K_{i,c}c_{i}-C_{i,p}\right)}{\frac{F}{RT}\sum_{i=1}^{n}z_{i}^{2}c_{i}}$$

The equilibrium suggested by Donnan is naturally ensured by applying Equation (10) to the two important interfaces, NF-PANZr/permeate solution and feed solution/NF-PANZr. This equilibrium is ensured by Equation (10) below:

(10) $$\frac{\gamma_{i}c_{i}}{\gamma_{i}{}^{0}C_{i}}=\Phi_{i}\exp\left(\frac{-z_{i}F}{RT}\Delta \Psi_{D}\right)$$

$z_{i}$, the valence of ion [i]; $\gamma_{i}^{0}$, bulk activity coefficient of ion [i], dimensionless; $\Delta \Psi_{D}$, Donnan potential variation (V); $\gamma_{i}$, activity coefficient of ion [i] within pore; T, temperature; $\Phi_{i}$, steric partition coefficient. Furthermore, the steric partition coefficient of ion i is obtained by Equation (11):

(11) $$\Phi_{i}={\left(1-\lambda_{i}\right)}^{2}$$

$\lambda_{i}$, the ratio of ionic solute radius $r_{i}$ to NF-PANZr pore radius, $r_{p}$. Considering an ideal condition, the steric partition has been removed from the Donnan equation. Assuming also that the solution is very dilute, the activity coefficient of ion [i] within the pore, to be taken into account inside the membrane by the effective charge density of the membrane, would be equal to 1. Equation (11) then becomes

(12) $$\left(\frac{c_{i}}{C_{i}}\right)=\exp\left(-\frac{z_{i}F}{RT}\Delta \Psi_{D}\right)$$

$c_{i},$ ionic concentration within pore, $\mathrm{mol}\cdot m^{-3}$; $\Delta \Psi_{D}$, Donnan potential variation (V); R, universal gas constant; F, Faraday constant; $C_{i}$, ionic concentration in the solution $\left(\mathrm{mol}\cdot m^{-3}\right)\;\mathrm{and}\;z_{i}$, valence of ion i;

The ion-i rejection (R) is given as follows:

(13) $$R=1-\frac{C_{i,p}}{C_{i,f}}$$

where $C_{i,f}$, the concentration of ion [i] in the feed solution $\left(\mathrm{mol}\cdot m^{-3}\right)$, $C_{i,p}$, ion-i concentration in the permeate $\left(\mathrm{mol}\cdot m^{-3}\right)$. Moreover, the ionic pore diffusion coefficient $D_{i,p}$ and the ionic hindrance factor for convection $K_{i,c}$ in the extended Nernst–Planck equation could be obtained respectively by Equations (14) and (15). The ionic pore diffusion coefficient, $D_{i,p}$, is then obtained as below:

(14) $$D_{i,p}=K_{i,d}.D_{i,\infty }$$

$K_{i,d}$, ionic hindrance factor for diffusion, dimensionless; $D_{i,p}$, the pore diffusion coefficient of ion [i], in $m^{2}\cdot s^{-1}$; $D_{i,\infty }$, ionic bulk diffusion coefficient (m^2^·s^−1^). The hindrance factor for convection $K_{i,c}$ is given, taking into account the ionic velocity in the nanofiltration membrane (NFM) pores [22]:

(15) $$K_{i,c}=\left(2-\Phi_{i}\right).G.\left(\lambda_{i},0\right)$$

$K_{i,c}$, ionic hindrance factor for convection; $\Phi_{i}$, ionic steric partition coefficient (Equation (11)); and G, hydrodynamic drag coefficient. The ionic hindrance factor for diffusion $K_{i,d}$ [25] is defined as follows:

(16) $$K_{i,d}=K^{-1}\left(\lambda_{i},0\right)$$

(17) $$G\left(\lambda_{i},0\right)=1.0+0.054\lambda_{i}-0.988\lambda_{i}^{2}+0.441\lambda_{i}^{3}$$

(18) $$K^{-1}\left(\lambda_{i},0\right)=1.0-2.30\lambda_{i}+1.154\lambda_{i}^{2}+0.224\lambda_{i}^{3}$$

$\lambda_{i}$, the ratio of the Stokes radius of element [i] to the pore radius of the membrane; G and K represent the hydrodynamic drag coefficients.

For any solute particle [i], the ratio of ionic or uncharged solute to the pore radius, which is dimensionless can be obtained by Equation (19) below.

(19) $$\lambda_{i}=\frac{r_{i}}{r_{p}}$$

where $r_{i}$ is the Stokes radius and $r_{p}$ is the effective pore radius (membrane) of ion i.

Equations (20) and (21) were finally obtained through a substitution of Equations (11) and (15)–(18) into Equations (5) and (9).

(20) $$\frac{dc_{i}}{dx}=\frac{J_{v}}{\left(1.0-2.30\lambda_{i}+1.154\lambda_{i}^{2}+0.224\lambda_{i}^{3}\right)D_{i,p}}\left[\left(2-{\left(1-\lambda_{i}\right)}^{2}\right)\left(1.0+0.054\lambda_{i}-0.988\lambda_{i}^{2}+0.441\lambda_{i}^{3}\right)c_{i}-C_{i,p}\right]\frac{z_{i}c_{i}}{RT}F\frac{d\Psi }{dx}$$

(21) $$\frac{d\Psi }{dx}=\frac{\sum_{i=1}^{n}\frac{z_{i}J_{v}}{\left(1.0-2.30\lambda_{i}+1.154\lambda_{i}^{2}+0.224\lambda_{i}^{3}\right)D_{i,p}}\left[\left(2-{\left(1-\lambda_{i}\right)}^{2}\right)\left(1.0+0.054\lambda_{i}-0.988\lambda_{i}^{2}+0.441\lambda_{i}^{3}\right)c_{i}-C_{i,p}\right]}{\frac{F}{RT}\sum_{i=1}^{n}z_{i}^{2}c_{i}}$$

These last two equations define the variation in concentration inside the active layer of NF-PANZr as a function of the effective pore-radius, $r_{p}$, and Stokes radius, $r_{i}$, of the ion (i).

### 2.3. Description of the Computation Procedure

The internal concentration, $c_{i,1}$, of ion [i] is relative to the feed-solution concentration, $C_{i,f}$, at the interface feed solution/membrane while the internal concentration, $c_{i,N}$, of ion (i) is relative to the permeate concentration, $C_{i,p}$, at the interface membrane/permeate solution. Runge–Kutta method was used to integrate both Equations (5) and (9) through the membrane active layer thickness $\Delta x=x_{2}-x_{1}$.

(i) Based on Equation (12), the knowledge of the value of $C_{i,f}$, makes possible the integration of both Equations (5) and (9) after the determination of the initial concentration inside the NF-PANZr membrane $\left(c_{i,1}\right)$.
(ii) Based on the Runge–Kutta numerical method, k_1_, k_2_, k_3_ and k_4_ and then $c_{i,1}$, $c_{i,2}$, $c_{i,3}$, $c_{i,4}$, …, $c_{i,N}$ could be well estimated (Equations (24)–(28); Equations (5) and (9)).
(iii) Since the $c_{i,N}$ value is obtained, the permeate concentration, $C_{i,p}$, was then computed.
(iv) Lastly, Equation (13) was used to evaluate the ion [i] rejection.

**Figure 1:**
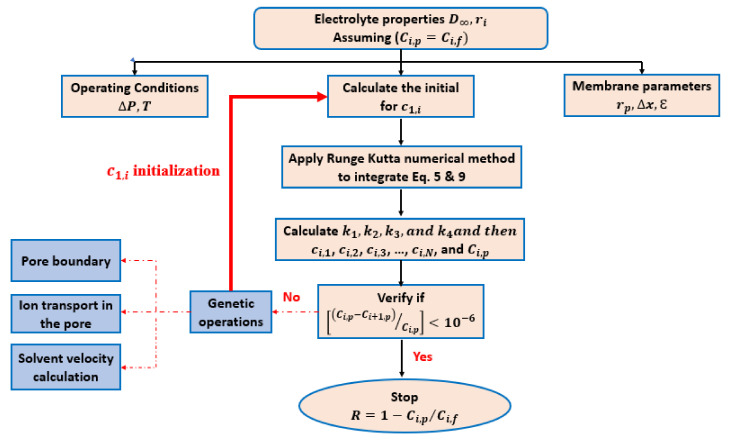
Flowchart describing the Runge–Kutta modeling of nanofiltration (NF) membrane transport equation.

The initial value for permeate solution, $C_{i,p}$, is assumed equal to the feed-solution concentration, $C_{i,f}$, which is the same as assuming zero rejection. The parameters such as hindered diffusivity, $D_{i,p}$; solute particle diffusivities; Stokes radii; the Donnan potential, ΔΨ_D_; partial molar volumes; and the hindrance factor for convection inside the membrane, $K_{i,c}$ can be found in the literature [26]. The membrane thickness, Δx; molecular weight cutoff (MWCO); and the membrane pore size, $r_{p};$ were available from the nanofiltration membrane synthesized in the present study. Figure 1 shows the flowchart describing the Runge-Kutta modeling of NF membrane transport equation.

### 2.4. Ion Transport across NF-PANZr Membrane

Equation (5) according to the Runge–Kutta numerical method was given as follows:

(22) $$\frac{c_{i,N+1}-c_{i,N}}{\Delta x}=\frac{J_{v}}{D_{i,p}}\left(K_{i,c}c_{i,N}-C_{i,p}\right)-\frac{z_{i}c_{i,N}}{RT}F\frac{d\Psi }{dx}$$

The step size is equal to the membrane thickness over the number $\left(n\right)$ of steps, n = 100.

(23) $$h=\frac{\Delta x}{n}$$

**Figure 2:**
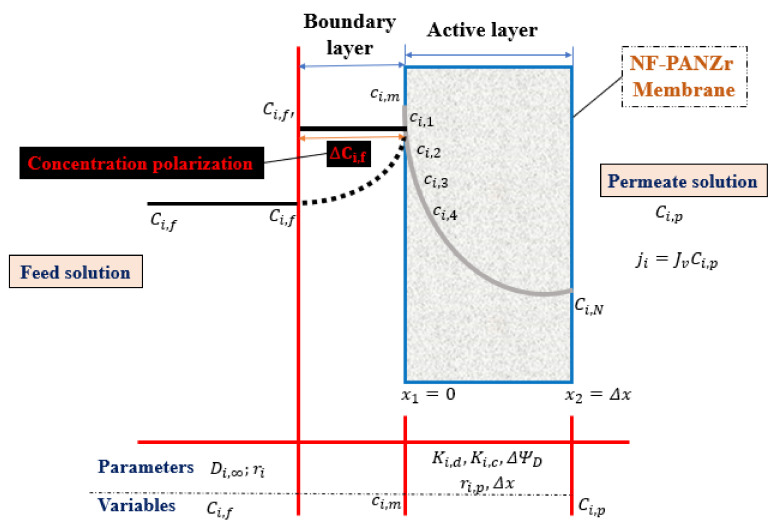
Solute particles transport across the novel synthesized NF-PANZr membrane active layer.

The active layer thickness ($\Delta x=x_{2}-x_{1}$) of the novel organic–inorganic NF-PANZr is depicted in Figure 2 while Table 1 showed the typical model parameters required, *h* is the step-size, n is equal to 100. The Runge–Kutta fourth-order formula, sometimes known as RK4, is given as

(24) $$k_{1}=hf\left(x_{i,n},c_{i,n}\right)$$

(25) $$k_{2}=hf\left(x_{i,n}+\frac{1}{2}h,c_{i,n}+\frac{1}{2}k_{1}\right)$$

(26) $$k_{3}=hf\left(x_{i,n}+\frac{1}{2}h,c_{i,n}+\frac{1}{2}k_{2}\right)$$

(27) $$k_{4}=hf\left(x_{i,n}+h,c_{i,n}+k_{3}\right)$$

(28) $$c_{i,n+1}=c_{i,n}+\frac{1}{6}k_{1}+\frac{1}{3}k_{2}+\frac{1}{3}k_{3}+\frac{1}{6}k_{4}+O(h^{5})$$

The solute particle [i] concentration inside the membrane active layer varies from $c_{i,1}$ at the feed solution/membrane interface side to $c_{i,100}$ at the membrane interface/permeate solution side. Thereafter, $c_{i,100}$ was used to calculate the permeate concentration, $C_{i,p}$.

(29) $$C_{i,p}=\frac{c_{i}}{\exp(-\frac{z_{i}F}{RT}\Delta \Psi_{D})}$$

The program, in this case, was kept running until the deviation between the initial and final permeate concentration would be inferior to $10^{-6}$, we can finally evaluate this difference, for verification, by using the last formula:

(30) $$Deviation=\frac{c_{i,p}-c_{i+1,p}}{c_{i,p}}$$

## 3. Experimental Section

### 3.1. Materials

Stacked flat sheets of a square section (1 m sides for each sheet) of UF polyacrylonitrile membrane possessing a molecular weight cutoff (MWCO) of 100 kDa purchased from SHANGHAI CORUN membrane technology Co., Ltd. (Shanghai, China). Various salts, ${\mathrm{CaCl}}_{2},{\mathrm{Na}}_{2}{\mathrm{SO}}_{4},\mathrm{NaCl},{\;\mathrm{MgSO}}_{4},{\mathrm{MgCl}}_{2}$ and zirconium sulfate tetrahydrate; deionized (DI) water; sodium bicarbonate buffer (${\mathrm{NaHCO}}_{3}$); and dopamine hydrochloride $\left(98\%,\;\mathrm{HCl}\cdot {\left(\mathrm{HO}\right)}_{2}C_{6}H_{3}{\mathrm{CH}}_{2}{\mathrm{CH}}_{2}{\mathrm{NH}}_{2}\right)$ were obtained from ALADDIN (Shanghai, China). Sodium hydroxide $\left(\mathrm{NaOH}\right)$ and hydrochloric acid $\left(\mathrm{HCl}\right)$ solution, and even ethanol $\left(C_{2}H_{5}\mathrm{OH}\right)$, were ordered from Sino Pharm Chemical Reagent Co.

### 3.2. Novel Organic–Inorganic Nanofiltration Membrane NF-PANZr Preparation

**Figure 3:**
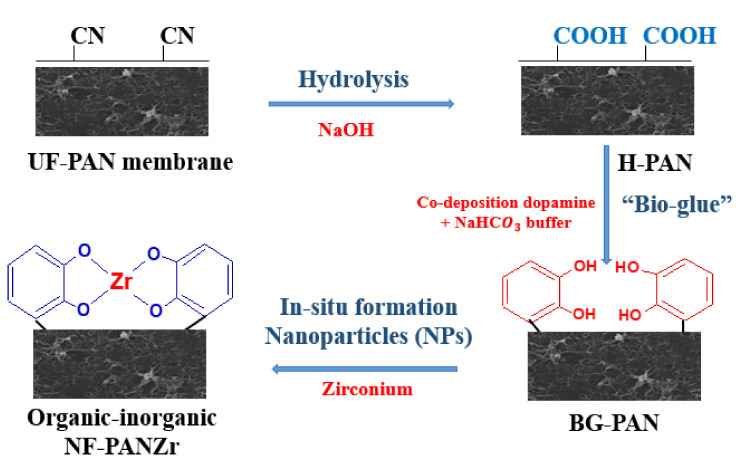
Procedure for novel thin-film composite membrane NF-PANZr synthesis.

We assume the different steps below for the synthesis of the NF-PANZr membrane. The main steps for NF-PANZr preparation are well depicted in Figure 3.

Step-1: Hydrolysis of polyacrylonitrile (PAN) membrane

UF membranes of polyacrylonitrile were hydrolyzed in a sodium hydroxide,NaOH, solution ($2.0\;\mathrm{mol}\cdot L^{-1}$) for 2 h at 50 °C. The resultant membrane is H-PAN.

Step-2: Co-deposition of dopamine hydrochloride (DA) and sodium bicarbonate buffer (Buffer)

A solution ($S^{1}$) was obtained from dopamine hydrochloride (DA) dissolved in a solution ($pH=8.0,\;50\;\mathrm{mmol}\cdot L^{-1}$ of sodium bicarbonate buffer (Buffer) for deposition. $S^{1}$ solution preparation is a result of in situ formation technology of DA–Buffer deposition on the PAN membrane surface in order to obtain a thin-film composite (TFC) layer acting like bio-glue. H-PAN membranes were pre-wetted using ethanol solution for 30 min before immersion into the solution ($S^{1}$) and stirred at 25 °C for 1 h 30 min. The resultant membranes (BG-PAN) were rinsed with pure water and dried in an oven set at 25 ± °C overnight.

Step-3: Deposition of zirconium (Zr) nanoparticles

In this last step of novel organic–inorganic thin-film composite nanofiltration synthesis, another fresh solution, $S^{2}$, is prepared by dissolving in hydrochloric solution ($50\;\mathrm{mmol}\cdot L^{-1}$), zirconium sulfate tetrahydrate with a concentration of $10\;\mathrm{mmol}\cdot L^{-1}$. Thereafter, the DA–Buffer coated H-PAN membrane pieces were immersed in the solution ($S^{2}$) at room temperature for 15 h. Following the deprotonation of the pyrocatechol groups ($C_{6}H_{4}{\left(\mathrm{OH}\right)}_{2}\to {\;C}_{6}H_{4}{\left(O-\right)}_{2}$) on the “bio-glue” layer, a covalent bond is established with Zr nanoparticles (as depicted in Figure 3) to generate a rigid layer of a complex compound of Zr. Finally, the resultant organic–inorganic NF-PANZr membranes were rinsed and dried in an ambient environment for later use and to be the subject of characterizations (properties, structures) and verification of performance.

### 3.3. NF-PANZr Membrane Properties’ Characterization

Water contact angle (WCA) measurements were performed with a Drop Meter A-200 contact angle system purchased from MAIST Vision Inspection & Measurement Co. Ltd., China. Measurements have been made for PAN, H-PAN, BG-PAN, and NF-PANZr membranes. These membranes were immersed in ethanol for 30 min and dried in an oven before the WCA was measured.

**Figure 4:**
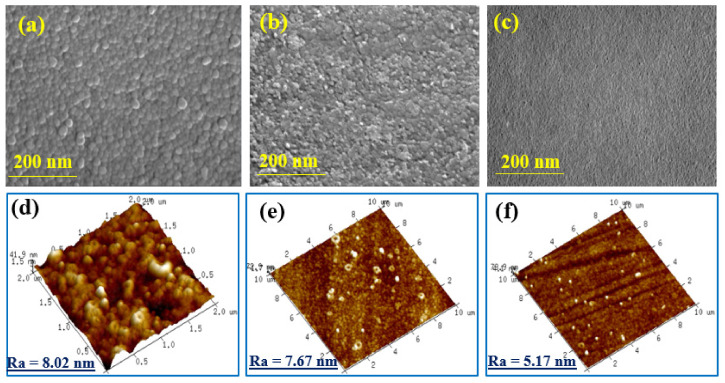
H-PAN (**a**,**d**); BG-PAN (**b**,**e**); and NF-PANZr (**c**,**f**) membranes’ field emission scanning electron microscopy (FESEM) and atomic force microscopy (AFM) images, respectively.

The electrokinetic analyzer purchased from SurPASS Anton Paar, GmbH, Austria was used for evaluating the charging property of the membranes’ surface. Four (04) samples had their zeta potential measured, PAN, H-PAN, BG-PAN, and NF-PANZr membranes as made explicit in Figure 4b.

The pore size distribution, effective mean pore size, and molecular weight cutoff (MWCO) of PAN, H-PAN, BG-PAN, and NF-PANZr membranes were measured by the solute particle rejection experiments using neutral rejection probes [27]. The feeds were glucose, sucrose, EG, α-CD, DEG, and PEG solutions of 150 ppm. Concentrations of organic solute particles were measured by a conductivity meter (Metrohm AG) and inductively coupled plasma optical emission spectroscopy (ICP-OES, Optima 7300 DV, PerkinElmer). The MWCO was calculated when the rejection reached 90%. The probability density function curve of the membrane was determined using the following Equation (31):

(31) $$\frac{dR(d_{p})}{d(d_{p})}=\frac{1}{d_{p}\sqrt{2\pi }\ln\sigma_{p}}\exp\left[-\frac{{\left(\ln d_{p}-\ln\mu_{p}\right)}^{2}}{2{\left(\ln\sigma_{p}\right)}^{2}}\right]$$

$d_{p}$ is the pore diameter. The mean pore size, ${µ}_{p}$, is the pore diameter at which rejection, *R*, is 50% and the geometric standard deviation, $\sigma_{p}=d_{p}\left(R=84.13\%\right)/d_{p}\left(R=50\%\right)$.

### 3.4. NF-PANZr Membrane Structure Characterization

Field emission scanning electron microscopy (FESEM) has been used for investigation on the surface morphology of the novel thin-film composite NF membrane, NF-PANZr created in this work.

Atomic force microscopy (AFM, Multi-Mode VECCO, Denton, TX, USA) was used to observe the morphology and roughness of H-PAN, BG-PAN, and NF-PANZr membranes, and the results are shown in Figure 4.

Energy dispersive spectrometer (EDS) was used together with FESEM in order to get more insights on the NF-PANZr surface elements’ arrangement, charge, and particularly about the phase state of the zirconium film. Zirconium (Zr), carbon (C), nitrogen (N), and oxygen (O) were among the different elements observed on the NF-PANZr surface.

### 3.5. Filtration Performance of Organic–Inorganic NF-PANZr Membrane

The performance of the novel organic–inorganic NF-PANZr has been investigated by a flat membrane module ensuring transverse flow at the laboratory scale under a pressure of 0.6 MPa, the temperature was set at 30 °C. Various classical salts ${\mathrm{Na}}_{2}{\mathrm{SO}}_{4},\mathrm{NaCl},{\mathrm{MgSO}}_{4},{\mathrm{CaCl}}_{2},{\;\mathrm{and}\;\mathrm{MgCl}}_{2}$ solutions were prepared at a concentration of $1000\;\mathrm{mg}\cdot L^{-1}$, using DI water, and used as feed solutions at a constant cross-flow rate of about $30\;L\cdot h^{-1}$ and the effective surface area of NF-PANZr samples was 29.22 cm^2^. The permeate flux ($J_{v},\;L\cdot m^{-2}\cdot h^{-1})$ and chemical rejection (R, %) were obtained using Equations (32) and (33):

(32) $$J_{v}=\frac{Q}{A\cdot t}$$

*A* is the NP-PANZr effective surface, *Q* is the permeate-solution volume, and *t* is the time of permeation across the NP-PANZr layer.

(33) $$R=\left(1-\frac{C_{p}}{C_{f}}\right)\times 100\%$$

$C_{f}$ and $C_{p}$ are, respectively, the solute particle concentration in the feed side and permeate side, $C_{p}$ and $C_{f}$ were determined by Metrohm AG, which is a conductivity meter, and another instrument, ICP-OES-Optima 7300 DV, PerkinElmer. All results presented were repeated at least three times, and the average values have been recorded and plotted.

### 3.6. Long-Term Stability of NF-PANZr Membrane

The NF-PANZr membrane was tested continuously for a whole week, and water flux and rejection readings were taken every 12 h and written down.

### 3.7. Richardson Extrapolation

Richardson’s extrapolation was used to get a better approximation of ${\mathrm{Cl}}^{-}{\;\mathrm{and}\;\mathrm{Mg}}^{2+}$ rejection. The Richardson extrapolation of order *n* is given by the formula:

(34) $$Q_{extra}≅\;\frac{2^{n}\times Q_{app}\left(\frac{h}{2}\right)-Q_{app}\left(h\right)}{2^{n}-1}$$

$Q_{app}\left(\frac{h}{2}\right)$ and $Q_{app}\left(h\right)$ are the Runge–Kutta model approximations’ values of experimental values respectively for n=200 and n=100 increments (steps); *h*, step size; *n*, number of steps.

### 3.8. Statistical Error Analysis

Error analysis was performed through the agreement between experimental and model data. In the *n* data points for each solute, the least-squares fitting objective function *RMSE* is defined as follows:

$$R^{2}=\frac{\sum_{i=1}^{n}{\left(Y_{exp,i}-Y_{model,mean}\right)}^{2}-\sum_{i=1}^{n}{\left(Y_{model,i}-Y_{exp,i}\right)}^{2}{}^{}}{\sum_{i=1}^{n}{\left(Y_{exp,i}-Y_{model,mean}\right)}^{2}}$$

$$RMSE=\sqrt{\frac{\sum_{i=1}^{n}{\left(Y_{model,i}-Y_{exp,i}\right)}^{2}}{n}}$$

where $Y_{exp}$ is experimental value, and $Y_{model,i}$ is predicted (model) rejection for ion i. Relative error (*RE*) was then calculated as, $RE=\frac{RMSE}{\bar{E}}$, $\bar{E}$ is the mean of experimental data. For a very good model, RE<0.1.

## 4. Results and Discussion

### 4.1. NF-PANZr Properties’ Characterization

**Figure 5:**
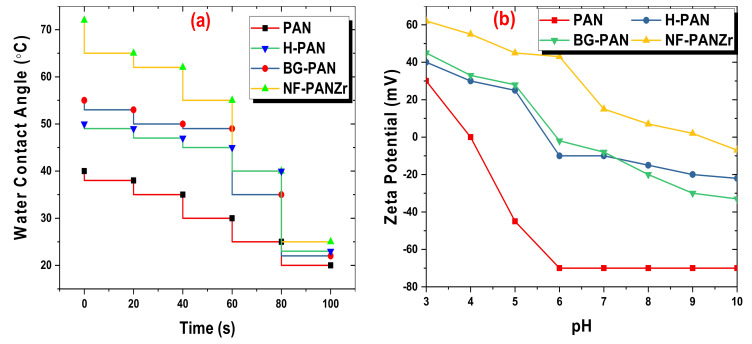
Polyacrylonitrile (PAN), hydrolyzed-PAN (H-PAN), BG-PAN, and NF-PANZr membranes (**a**) water contact angle and (**b**) zeta potential.

The evaluation of the wettability of PAN, H-PAN, BG-PAN, and NF-PANZr membranes, performed with a Drop-Meter A200 contact angle system purchased from MAIST Vision Inspection & Measurement Co., Ltd., China, is shown in Figure 5a. The PAN membrane exhibited the best hydrophilicity since it possesses the most porous structure. The water contact angles of H-PAN and BG-PANZr were quite similar mainly from 60 s. The WCA has increased with the deposition of Zr nanoparticles (NPs). Although the novel organic–inorganic NF-PANZr membrane is the least hydrophilic of the four membranes tested in this section, it exhibits excellent hydrophilicity since the water contact angle is less than 72 °. Therefore, the water molecules could quickly spread over the NF-PANZr surface before seeping in.

The pseudo-measurement of the Donnan potential was used to determine the membrane surface zeta potential (ζ) as shown in Figure 5b [28,29,30]. The state of PAN, H-PAN, BG-PAN, and NF-PANZr membranes’ surface charge was determined for pH ranged between 3 and 10. BG-PAN membrane (bio-glue coated the PAN substrate) exhibited a negatively charged surface in contact with a basic solution (pH > 6), due to the deprotonation of the pyrocatechol groups ($C_{6}H_{4}{\left(\mathrm{OH}\right)}_{2}\to {\;C}_{6}H_{4}{\left(O-\right)}_{2}$) on its top layer surface. The NF-PANZr membrane surface is positively charged unlike the BG-PAN membrane surface as a result of the deposition of Zr NPs that in solution generate positive ions ${\mathrm{Zr}}^{4+}$. The NF-PANZr membrane is positively charged in the pH range used to carry out the experiments, namely 6±0.5. Furthermore, the more or less attractive or repulsive effect of the NF-PANZr membrane on ion rejection was highlighted in the analysis of the results produced by the Runge–Kutta model. The charge of the membrane plays a very important role in the transport of ions through the membrane active layer, and several recent studies confirm this propensity of the membrane to attract and allow itself to be more easily crossed by ions of a charge contrary to its charge [8,31,32,33,34].

The four membranes showed a negative charge above pH 9. The negative ions could then be easily and efficiently rejected for pH > 9 according to the Donnan effect. If the NF-PANZr membrane was used to remove ions at high pH (pH > 9), its behavior depicted above toward negatively charged ions will change. Instead of an attraction, we will expect a repulsion of these ions. Thus, for two ions, taken under the same stoichiometric conditions and of the same valence, the organic–inorganic NF-PANZr will exhibit better rejection toward the negatively charged ion. However, several parameters govern and condition the transport of ions.

### 4.2. NF-PANZr Structures’ Characterization

The H-PAN, BG-PAN, and NF-PANZr membranes’ surface morphology and roughness, which are valuable features in NF are depicted in Figure 4. The H-PAN membrane surface is porous (Figure 4a) and accordingly, its roughness is the highest as shown in the AFM image (Figure 4d). The “bio-glue” (dopamine hydrochloric–sodium bicarbonate buffer) coated substrate generated a smoother surface with almost invisible pores as depicted in Figure 4b,e. The hydrolyzed polyacrylonitrile membrane (H-PAN), once modified by deposition of zirconium nanoparticles displays the most smooth surface without visible pores (Figure 4c), while the membrane roughness is very small according to the AFM image shown in Figure 4e.

The membrane roughness decreased, to reach Ra = 5.17 nm, for the novel thin composite NF-PANZr membrane. The DA–Buffer coated the polyacrylonitrile substrate that acts like “bio-glue” has played an important role in zirconium NPs’ deposition, having made it possible to obtain a more uniform and thinner membrane surface. Lv et al. have concluded from a recent study performed on a TFC NF membrane that a dense and smooth selective layer is beneficial for excellent rejection performance [35]. The NF-PANZr membrane may, therefore, provide an excellent solute particle removal and high permeation performance. This assertion will be verified later in this investigation.

**Figure 6:**
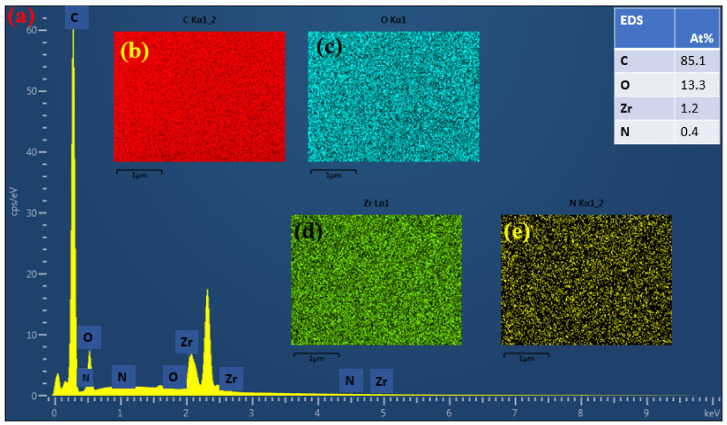
(**a**) Energy spectrum analysis results of the NF-PANZr and the sample table surface elements containing (**b**) carbon, (**c**) oxygen, (**d**) zirconium, and (**e**) nitrogen.

To hold more insights into the arrangement of the Zr nanoparticle (NP) layer and its potential function on the membrane platform, the energy dispersive spectrometer (EDS) was associated with the field emission scanning electron microscopy (FESEM). Figure 6 shows the energy spectrum irradiation of elements on the NF-PANZr membrane surface. Four different elements, including nitrogen (N), carbon (C), oxygen (O), and zirconium (Zr) were displayed by the energy dispersive spectrometer image results, due to their unique X-ray signals. Zr, N, O, and C elements were distributed uniformly on the surface of the novel synthesized NF-PANZr membrane as shown in Figure 6b–e, respectively. It is therefore possible now to modify and characterize the materials at the atomic scale, providing unparalleled insight into the behavior of nanomaterials and particles, since each atomic position can be distinctly distinguished by its specific chemical signal (Figure 6a). The polyacrylonitrile used for the preparation of the NP-PANZr membrane is an organic membrane; carbon is, therefore, the most dominant element of the new membrane and consequently, a very strong signal is observed at the element C position. The sequence of the presence of the elements is C > O > Zr > N.

**Figure 7:**
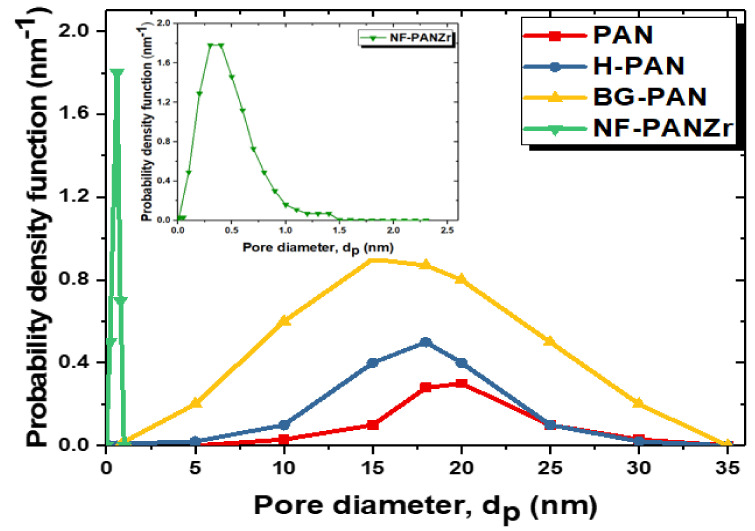
Probability density function curves of the PAN substrate, H-PAN, BG-PAN, and NF-PANZr membranes.

Figure 7 shows the pore size distribution curves of the PAN substrate, and H-PAN, BG-PAN, and NF-PANZr membranes, while their properties are reported in Table 2 as a function of copolymer content. The PAN substrate exhibits the largest pore diameter of about 20 nm. After the in situ formation process, the pore diameter of the resultant membrane (NF-PANZr) decreases dramatically to 0.4 nm. The hydrolysis step influenced the polymer very slightly since its pore diameter was around 18 nm. The observation is the same for the step of covering the membrane with DA/Buffer. On the other hand, the Zr nanoparticles significantly modified the membrane in terms of its structure. Figure 5 and Figure 7 are a good match. The nanoparticles have contributed to significantly reduce the pores of the PAN membranes, which served as a platform (Figure 7). These nanoparticles are fine elements that have helped to close the “voids” of the PAN membrane, which, being an ultrafiltration membrane, is more porous. This resulted in a smoother structure with invisible pores (Figure 4).

### 4.3. Experimental Salt Rejection

**Figure 8:**
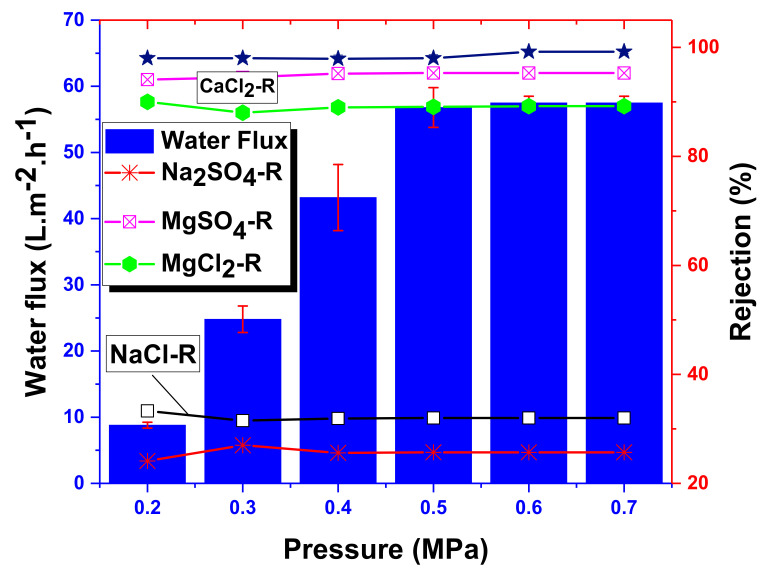
Water flux and salt rejection of NF-PANZr as a function of transmembrane pressure under conditions, $\left[Pressure\right]\;=\;0.6\;\mathrm{MPa}$, $\left[Temperature\right]\;=\;25\;{}^{\circ}C$, $[Salts]\;=\;1000\mathrm{mg}\cdot L^{-1}$, pH = 6±0.5.

The water flux and salt rejection of the NF-PANZr membrane are shown in Figure 8 for various salt solutions including $\mathrm{NaCl},{\mathrm{MgSO}}_{4},{\mathrm{Na}}_{2}{\mathrm{SO}}_{4},{\mathrm{CaCl}}_{2},{\mathrm{and}\;\mathrm{MgCl}}_{2}$ at different applied transmembrane pressures, ΔPe = 0.2, 0.3, 0.4, 0.5, 0.6, and 0.7 MPa. The water flux only increases with the pressure until it stabilizes around $58\;L\cdot m^{-2}\cdot h^{-1}$ under a pressure of 0.5 MPa. The thin-film composite NF-PANZr exhibited an excellent rejection performance for ${\mathrm{MgCl}}_{2},{\mathrm{CaCl}}_{2},{\mathrm{and}\;\mathrm{MgSO}}_{4}$ salts. These salts’ rejection reached, respectively, 89.2%, 99.3%, and 95.3%. ${\mathrm{Na}}_{2}{\mathrm{SO}}_{4}\;\mathrm{and}\;\mathrm{NaCl}$ salts were not efficiently removed by the novel synthesized membrane NF-PANZr. The salts’ rejection sequence can be summarized as ${\mathrm{CaCl}}_{2}>{\mathrm{MgSO}}_{4}>{\mathrm{MgCl}}_{2}>\;\mathrm{NaCl}>{\mathrm{Na}}_{2}{\mathrm{SO}}_{4}$.

As a result, NF-PANZr has shown its ability to effectively reject multivalent ions such as ${\mathrm{Mg}}^{2+},{\;\mathrm{Ca}}^{2+},{\mathrm{and}\;\mathrm{SO}}_{4}^{2-}$ from water and its incapability to remove monovalent ions such as ${\mathrm{Cl}}^{-}$. This point of view is widely shared by a large number of publications recently performed [36,37,38]. Table 3 below provides an overview of the data used in the present model.

### 4.4. Runge–Kutta Model Reevaluation of Cl^−^ and Mg^2+^ Rejection

**Figure 9:**
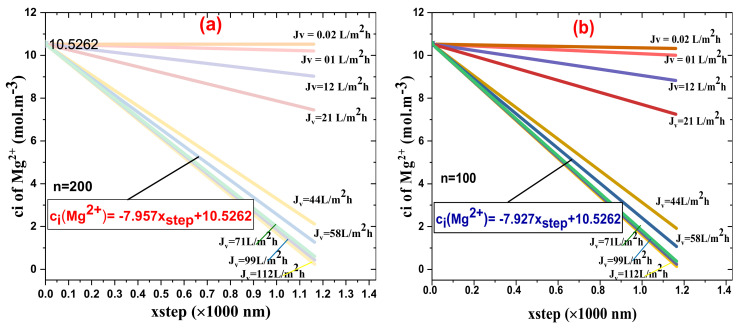
Concentration of the ion ${\mathrm{Mg}}^{2+}$ inside the novel synthesized nanofiltration membrane (NFM), NF-PANZr, active layer for different volumetric flux ($J_{v}$ ) as a function of step size: (**a**) number of steps equals 200, (**b**) number of steps equals 100.

Figure 9a,b exhibits the Mg^2+^ ion concentration inside the novel synthesized NFM, NF-PANZr, active layer for different volumetric flux (*J_v_*) as a function of step size, Figure 9b is for *n* = 100, while Figure 9a represents the plots for number of steps = 200.

In both figures, the concentration of the divalent cation Mg^2+^ in the permeate decreases dramatically for the highest water flux ( *J_v_* > 40 L · m^−2^ · h^−1^), especially in the typical cases of number of steps = 200. Globally, the divalent cation Mg^2+^ ions in the permeate decrease with increasing permeate flux *J_v_* (L · m^−2^ · h^−1^), and this occurs independently of the number of incrementation steps. On the contrary, the rejection of Mg^2+^ ions increased as the water flux (*J_v_*) through the NF membrane increased. NF-PANZr rejection of Mg^2+^ in the typical case of *n* = 200 was higher than that of *n* = 100. A smaller and more refined increment step, therefore, promotes the rejection efficiency. In the hypothetic case of *J_v_* = 58 L · m^−2^ · h^−1^ (salt MgSO_4_) the theoretical rejection of the Mg^2+^ ion is, respectively, 95.1% and 91.8% for *n* = 200 and *n* = 100. Since the experimental rejection of Mg^2+^ is 95.3%, the relative error made in the typical case where a high number of increments has been chosen is very closed to 0.02% (Table 4).

Moreover, Figures S1 and S2 depict the ${\mathrm{SO}}_{4}^{2-}$ ion concentration inside the membrane active layer versus the step size for different water flux, $J_{v}$.

NF-PANZr rejection of ${\mathrm{Mg}}^{2+}$ was higher than the rejection of ${\mathrm{SO}}_{4}^{2-}$. Such rejection behavior is related to the membrane charge, which is of a positive charge at pH 6 (Figure 5b). Figure 3 shows the deposition of the ion ${\mathrm{Zr}}^{4+}$ on the membrane surface.

From Equations (6) and (8), the NF-PANZr charge effect appeared in the module in the electrical potential gradient and resulted in a disparate influence on ${\mathrm{SO}}_{4}^{2-}{\;\mathrm{and}\;\mathrm{Mg}}^{2+}$ removal. Repulsion between the NF-PANZr membrane charge and the ${\mathrm{Mg}}^{2+}$ ions occurred while the attraction between the NF-PANZr membrane charge and the ${\mathrm{SO}}_{4}^{2-}$ ions took place; in other words, ${\mathrm{SO}}_{4}^{2-}$ ions would pass more freely across the membrane active layer, while the ${\mathrm{Mg}}^{2+}$ ions would be pushed back. For all plots, it was noticed that the concentration of ${\mathrm{SO}}_{4}^{2-}$ and ${\mathrm{Mg}}^{2+}$ ions inside the membrane active layer decreased as the ions moved via the membrane active layer from the feed side to the permeate side.

Another parameter that affected ${\mathrm{SO}}_{4}^{2-}$ and ${\mathrm{Mg}}^{2+}$ ions’ rejection is the membrane active layer pore size, $r_{p}$, which appears in the hindrance factor for diffusion, $K_{i,d}$, and the hindrance factor for convection, $K_{i,c}$, as clarified in Equations (9), (14), and (15). In the typical case of a water flux, $J_{v}$, of 58 L · $m^{-2}\cdot h^{-1},$ the concentrations of ${\mathrm{Mg}}^{2+}$ ions across the NF-PANZr membrane can be determined for number steps (100 and 200) by the respective equations:

$$c_{i}\left(Mg^{2+}\right)=-\;7.927\times x_{step}+10.5262;\;n=100;\;c_{i}\left(Mg^{2+}\right)=-\;7.957\times x_{step}+10.5262;\;n=\;200.$$

**Figure 10:**
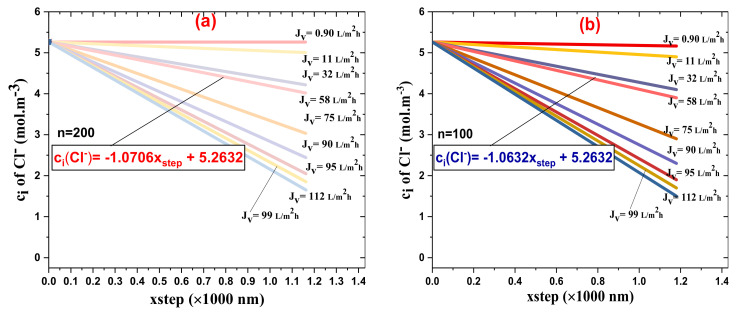
Concentration of the ion ${\mathrm{Cl}}^{-}$ inside the novel synthesized NFM, NF-PANZr active layer for different volumetric fluxes ($J_{v}$) as a function of step size: (**a**) number of steps equals 200, (**b**) number of steps equals 100.

Figure 10a,b depicts the ${\mathrm{Cl}}^{-}$ ion concentration inside the membrane active layer as a function of the step size for different water fluxes, $J_{v}$; Figure 10a stands for n = 200 while Figure 10b is for n=100.

Globally, the ${\mathrm{Cl}}^{-}$ ions’ rejection increased as the water flux, $J_{v}$, via the NF-PANZr membrane increased. Since the thickness ($x_{2}-x_{1})$ of the membrane has been divided by 100 (Figure 10b) and then by 200, the *xstep* has been set at 11.8 nm in the first case and 5.9 nm in the second case. NF-PANZr rejection of ${\mathrm{Cl}}^{-}$ was higher for a small step size (Figure 10a) for all water fluxes, $J_{v}$. In the hypothetic case of $J_{v}=\;58\;L\cdot m^{-2}\cdot h^{-1}$, the theoretical rejection of the ${\mathrm{Cl}}^{-}$ ion is, respectively, 32.2% and 36.4% for *n* = 200 and *n* = 100. Since the experimental rejection is 31.9%, the relative error made in the typical case where a high number of increments has been chosen is small.

To better analyze the behavior of the NF-PANZr membrane concerning the monovalent ions ${\mathrm{Cl}}^{-}{\;\mathrm{and}\;\mathrm{Na}}^{+}$ taken under the same stoichiometric conditions, Figures S3 and S4 show the plots of ion ${\mathrm{Na}}^{+}$ model rejection for *n* = 200 and *n* = 100 respectively. NF-PANZr rejection of ${\mathrm{Na}}^{+}$ was higher than the rejection of ${\mathrm{Cl}}^{-}$. Such rejection behavior is related to the membrane charge, which is of a positive charge at pH 6 (Figure 5b). Figure 3 shows the deposition of the ion ${\mathrm{Zr}}^{4+}$ on the membrane surface.

From Equations (6) and (8), the NF-PANZr charge effect appeared in the module in the electrical potential gradient and resulted in a disparate influence on ${\mathrm{Cl}}^{-}{\;\mathrm{and}\;\mathrm{Na}}^{+}$ removal. Repulsion between the NF-PANZr membrane charge and the ${\mathrm{Na}}^{+}$ ions occurred, while the attraction between the NF-PANZr membrane charge and the ${\mathrm{Cl}}^{-}$ ions took place; in other words, ${\mathrm{Cl}}^{-}$ ions would pass more freely across the membrane active layer, while the ${\mathrm{Na}}^{+}$ ions would be pushed back. For all plots shown in Figure 10a,b and Figures S3 and S4, it was noticed that the concentration of ${\mathrm{Cl}}^{-}$ and ${\mathrm{Na}}^{+}$ ions inside the membrane active layer decreased as the ions moved via the membrane active layer from the feed side to the permeate side. The Table 5 depicts the properties such as Stokes radii, diffusivities, and partial molar volumes of solute particles used in this investigation.

Another parameter that affected ${\mathrm{Cl}}^{-}$ and ${\mathrm{Na}}^{+}$ ions’ rejection is the membrane active layer pore size, $r_{p}$, which appears in the hindrance factor for diffusion, $K_{i,d}$, and the hindrance factor for convection, $K_{i,c}$, as clarified in Equations (9), (14) and (15).

In the typical case of a water flux, $J_{v}$, of 58 L·$m^{-2}\cdot h^{-1},$ the concentrations of ${\mathrm{Cl}}^{-}$ ions across the NF-PANZr membrane can be determined for number steps = 100 and 200 by the respective equations:

$$c_{i}\left(Cl^{-}\right)=-\;1.0632\times x_{step}+\;5.2632;\;n=100;\;c_{i}\left(Cl^{-}\right)=-\;1.0706\times x_{step}+\;5.2632;\;n=200.$$

### 4.5. NF-PANZr Long-Term Stability

**Figure 11:**
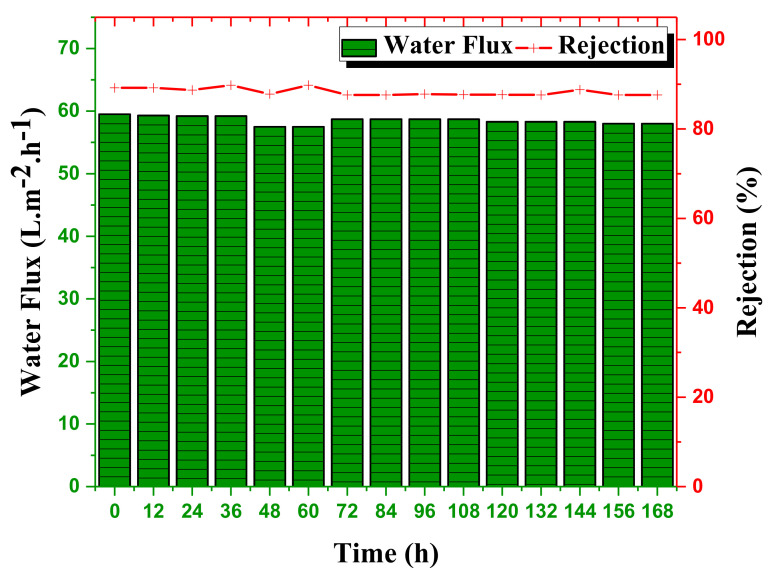
Test of long-term stability carried out on the synthesized organic-inorganic NF-PANZr membrane under test conditions: $\mathrm{cross}-\mathrm{flow}\;\mathrm{rate}\;=\;30\;L\cdot h^{-1}$; pH=6; [${\mathrm{MgCl}}_{2}]\;=\;1000\;\mathrm{mg}/L$, 168 h test; pressure = 0.6 MPa.

The long-term stability of the novel organic–inorganic NF-PANZr membrane was investigated under a continuous filtration test for one week, and the results are shown in Figure 11. Of the various salts studied in this work, the long-term stability test was performed with ${\mathrm{MgCl}}_{2}$.

The conditions under which the experiment was carried out were as follows: pH = 6.0, concentration $\left[\mathrm{MgCl}\right]$ 1000 mg/L, temperature = 30 °C, transmembrane pressure ΔPe=0.6 MPa, cross − flow rate = $30\;L\cdot h^{-1}$. Globally, the thin-film composite NF membrane NF-PANZr has shown excellent long-term stability for permeate flux and salt removal. The water flux through the NF-PANZr membrane was almost constant over time for a slight decrease of less than 1.1%. The water flux was about $58\;L\cdot m^{-2}\cdot h^{-1}$ during the long-term operation and did not change remarkably till the end of the test. During the 168 h test, the novel organic–inorganic NF membrane exhibited an excellent rejection performance of 88.9% toward ${\mathrm{MgCl}}_{2}$ salt. The rejection, like water flux, hardly varied over time, which is the advantage of this newly synthesized membrane. This one-week continuous test made it possible to appreciate the long-term effectiveness of the NF-PANZr membrane synthesized in this work. The good durability of the NF-PANZr membrane is closely related to the interfacial compatibility between the ${\mathrm{ZrO}}_{2}$ nanoparticles (NPs) as a selective layer, the support surface polyacrylonitrile (PAN) membrane through the robust and multiple binding forces between the DA–Buffer (“bio-glue”) coating and the H-PAN membrane.

### 4.6. Richardson Extrapolation and Statistical Error Analysis

The results predicted by the Runge–Kutta method are very far from the experimental results if the number of incrementations chosen is *n* = 100. However, these results are very good in the case where the number of incrementations is large, n = 200, and consequently produces a very small step size, $Q_{\mathrm{app}}\left(h/2\right)$. Using Richardson’s extrapolation, the theoretical results are found to be very refined and practically the same as the experimental ones.

### 4.7. Comparison of the Model Implemented in This Study with Other Previous Membrane Models

Most of the modeling-based research has been carried out on commercial membranes. The only reported work that combines a synthesized membrane and modeling, to our knowledge, is that of Farci et al. [39]. This famous work reported a Donnan steric-pore model with dielectric exclusion (DSPM-DE) model for both water flux and salt rejection prediction of microporous organosilica and mesoporous γ-alumina membranes, and scanning electron microscopy (SEM) was used to check the structure of the modified membrane. Table 6 provides an overview of some work on modeling.

This work reports a novel NF membrane obtained from the deposition of Zr nanoparticles; the NF-PANZr membrane is fully characterized from a structural point of view (FESEM, EDS, AFM images), and its properties have been amply elucidated (water contact angle and zeta potential). The Runge–Kutta method supplemented by Richardson’s extrapolation has been shown to be very effective in predicting ion rejection. This fact is understandable when we compare the numerical method of Euler (RE=0.9%) to that of Runge–Kutta (the numerical method of Euler is the numerical method of first-order Runge–Kutta). The fourth order Runge–Kutta has been refined by the Richardson extrapolation. The nature and specificity of the project on which we are working require it.

## 5. Conclusions

The NF-PANZr membrane, like the NF membranes, has demonstrated excellent rejection performance against multivalent ions, and the ion rejection prediction model based on the Runge–Kutta method has been shown to be effective. In addition, the long-term stability carried out on NF-PANZr reassured that the membrane can be used for a long time before being replaced, especially if the membrane cleaning work consisting of its bombardment with drafts is periodically undertaken. The models are set up and calibrated for predefined input parameters, and they are not subject to change as chemical agents are changed on an experimental scale. We strongly recommend, therefore, that the scientific world study the distinctive characteristics of the next-generation NF membranes by incorporating the use of simple but effective models for more reliable results.

Further validation of existing models and the development of better NF models is a requirement for better NF membrane characterization, perfect chemical rejection reassessment, and even more insights on additional experimental available data. Beyond physical models, the rise of machine learning in the prediction of the rejection performance of NF membranes is also a horizon to explore.

## Figures and Tables

**Table 1:** A typical set of model parameters used in the computation.

Parameters	Abbreviation	Value
Faraday’s constant (F)	F	96,487 C·mol^−1^
Universal gas constant	R	8.314 $J\cdot {\mathrm{mol}}^{-1}\cdot K^{-1}$
Boltzmann constant (k)	K	1.38066 × 10^−23^ J·K^−1^
Permittivity of free space ($\varepsilon_{0})$	$\varepsilon_{0}$	8.85419 $\times 10^{-12}\;J^{-1}\cdot C^{2}\cdot m^{-1}$
Operating temperature (T)	T	303.15 K
Operating pressure ($\Delta P_{e}$)	P	0.60 MPa
Hydrogen potential	pH	6.0
Crossflow velocity	CFV	$30\;L\cdot h^{-1}$

**Table 2:** Mean effective pore diameter ${µ}_{p}$; molecular weight cutoff (MWCO); and geometric standard deviation, $\sigma_{p}$.

Membrane	$\mu_{p}(nm)$	$\sigma_{p}$	MWCO (kDa)
**PAN Platform**	21	1.13	100
**H-PAN**	18	1.32	13
**BG-PAN**	15	1.36	17
**NF-PANZr**	0.4	1.45	8.8

**Table 3:** Other membrane characteristics and filtration parameters used in this study.

Parameters	Units	Value	References
Rejection_NaCl	%	32.0	This study
Rejection_MgSO_4_	%	95.3	
Permeate_flux	$L\cdot m^{-2}\cdot h^{-1}$	58	
Membrane_geometry	Flat − Sheet	1 m × 1 m	
Membrane_surface area	cm^2^	29.22	
Membrane_thickness	nm	1180 ± 5.17	
Pore_size	nm	0.4	Equation (31)

**Table 4:** Experimental and predicted rejections followed by error estimate.

	NF-PANZr Membrane				
	Experimental	Predicted			Error (%) RE
		$Q_{app}\left(h/2\right)$	$Q_{app}\left(h\right)$	$Q_{extra}$	
${\mathrm{Cl}}^{-}$	31.9	32.2	36.2	31.93	0.09
${\mathrm{Mg}}^{2+}$	95.3	95.1	92.3	95.29	0.01

**Table 5:** Diffusivities, Stokes radii, and partial molar volumes of ions used in this study.

Ion	Ion Diffusivity $\left(D_{\infty },10^{-11}m^{2}\cdot s^{-1}\right)$	Stokes Radii $\left(r_{i},\;nm\right)$	Partial Molar Volume $\left(V_{i},\;cm^{3}\cdot mol^{-1}\right)$	References
$Na^{+}$	133	0.184	−1.20	[22]
$Cl^{-}$	203	0.121	17.82	
$SO_{4}^{2-}$	106	0.231	14.18	
$Mg^{2+}$	72	0.348	−21.57	

**Table 6:** NF models performed in this study and in other previous investigations.

Membrane Type	Designation	Model	Error (%)	References
**Synthesized**	NF-PANZr	Runge–Kutta + Richardson Extrapolation	0.01–0.09	This study
	γ− alumina mesoporous	DSPM−DE model	0.05	[39]
	Organosilica microporous	DSPM−DE model	<0.05	
	NF_PAN_TI	Euler numerical method	0.09	
**Commercial**	NF 90	Gauss–Newton	1.91	[40]
	NF 270	Gauss–Newton	3.34	
	NF-1	DSPM modified	1.2	[41]
	NF-2	DSPM modified	5.2	
	NF-20	DSPM modified	3.4	
	UTC-70UB	GP model	0.20	[42]
	Desal-DK	2P model + dielectric	0.30	[22]
	Desal-DK	DSPM	0.13	

## Data Availability

No new data were created or analyzed in this study. Data sharing is not applicable to this article.

## Supplementary Materials

The following are available online at https://www.mdpi.com/2077-0375/11/2/130/s1, Figure S1: Concentration of ion $SO_{4}^{2-}$ inside the novel synthesized NFM, NF-PANZr active layer for different volumetric flux ($J_{v}$) as a function of step-size—Number of steps equals 200, Figure S2: Concentration of ion $SO_{4}^{2-}$ inside the novel synthesized NFM, NF-PANZr active layer for different volumetric flux ($J_{v}$) as a function of step-size—Number of steps equals 100, Figure S3: Concentration of ion $Na^{+}$ inside the novel synthesized NFM, NF-PANZr active layer for different volumetric flux ($J_{v}$) as a function of step-size—Number of steps equals 200, Figure S4: Concentration of ion $Na^{+}$ inside the novel synthesized NFM, NF-PANZr active layer for different volumetric flux ($J_{v}$) as a function of step-size—Number of steps equals 100.

## Nomenclature

$c_{i}$	ion [i] concentration within pore, mol·m^−3^
$C_{f}$	feed-solution concentration, mol·m^−3^
$C_{i}$	ion [i] in feed-solution concentration, mol·m^−3^
$C_{p}$	uncharged solute bulk permeate concentration, mol·m^−3^
$D_{p}$/ $D_{i,p}$	uncharged solute/charged solute pore diffusion coefficient, $m^{2.}s{-}^{1}\;(=K_{d}D_{\infty })$
$D_{\infty }$	solute bulk diffusion coefficient, m^2.^s^−1^
e	electronic charge, $1.602177\times 10^{-19}C$
I	ionic strength, mol·m^−3^
j	number of data points per solute in fitting, dimensionless
$j_{i}$	ionic flux of ion [i] (pore area basis), mol·m^−2^·s^−1^
$j_{s}$	uncharged solute flux (pore area basis), mol·m^−2^·s^−1^
k	feed-side mass transfer coefficient, m/s
k	Boltzmann constant, $1.38066\times 10^{-23}{\mathrm{JK}}^{-1}$
$K_{i,c}$	hindrance factor for convection of ion I, dimensionless
$K_{i,d}$	ionic hindrance factor for diffusion, dimensionless
P	pressure N/m^2^
$r_{p}$	effective pore radius, m
R	rejection (%)
R	universal gas constant, $8.314\;J\cdot {\mathrm{mol}}^{-1}\cdot K^{-1}$
V	solvent velocity, m/s
$X_{d}$	effective charge density, mol/m^3^
T	absolute temperature in K
$z_{i}$	ion [i] valence, dimensionless
x	axial position within the pore, m
$\gamma_{i}$	the activity coefficient of ion [i] within the pore, dimensionless
ΔP	applied pressure, N·m^−2^
$\gamma_{i}^{0}$	the bulk activity coefficient of ion [i], dimensionless
$\Delta P_{e}$	effective pressure driving force, N·m^−2^
Δx	membrane thickness, m
ΔΠ	the osmotic pressure difference, N·m^−2^
λ	the ratio of ionic or uncharged solute radius to pore radius, dimensionless
$\Delta \Psi_{D}$	Donnan potential, V
η	solvent viscosity within pores, N·s·m^−2^
εbεp	bulk/pore dielectric constant, dimensionless
ξ	the ratio of effective membrane charge density to bulk feed concentration, dimensionless $(\xi =X_{d}/C_{f})$
$\lambda_{i}$	the ratio of ionic radius to pore radius, dimensionless
$\Phi_{i}$	the steric partition coefficient of ion [i], dimensionless
Ψ	the potential within the pore, V
