# Supplementary material for: Runge–Kutta Numerical Method Followed by Richardson’s Extrapolation for Efficient Ion Rejection Reassessment of a Novel Defect-Free Synthesized Nanofiltration Membrane
Source: Membranes (Basel). 2021 Feb 14;11(2):130. doi: 10.3390/membranes11020130 (PMC7918593; doi:10.3390/membranes11020130)
Supplement: Supplementary file 1 [file membranes-11-00130-s001.pdf]

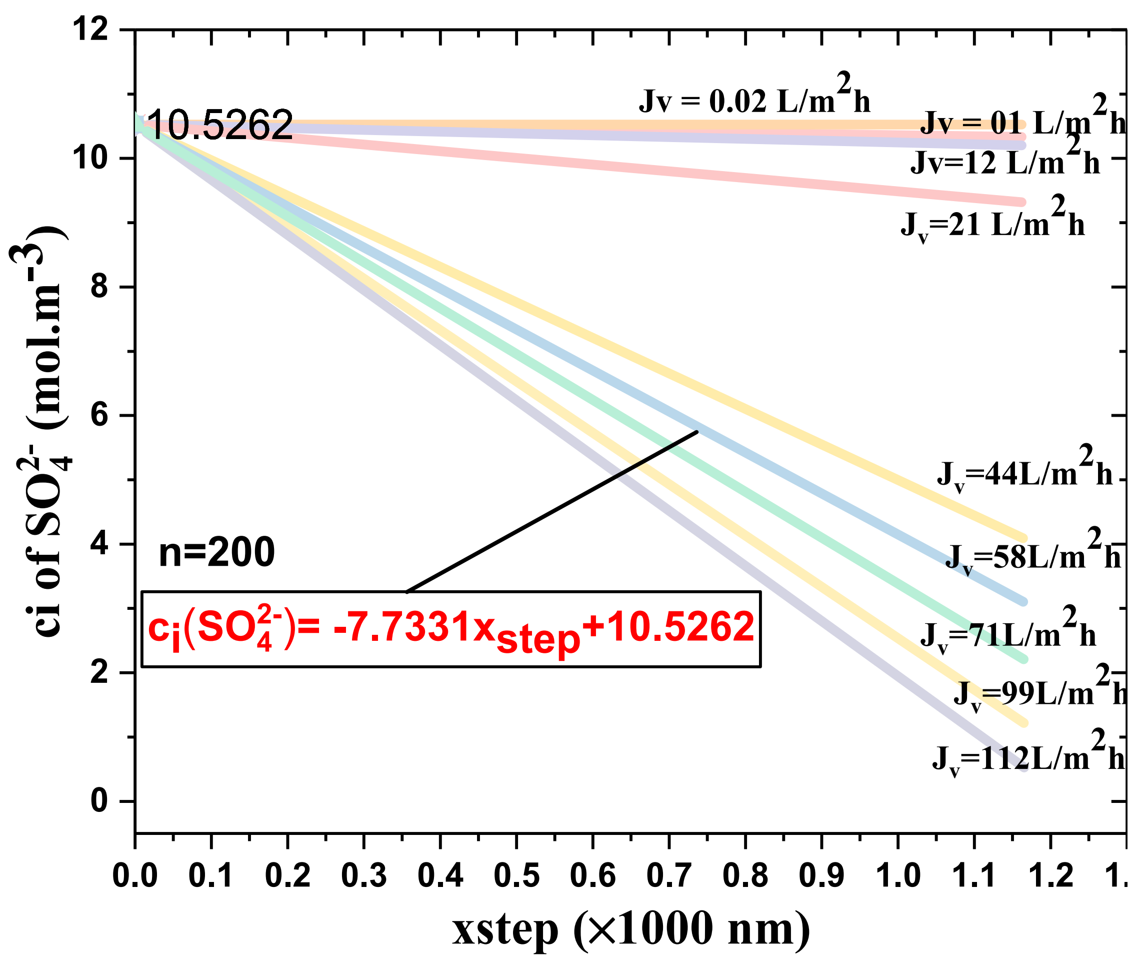

Figure S1: SO_4^{2-} ion concentration inside the membrane active layer versus the step-size (for different volumetric flux (J_v)) – Number of steps equals 200.

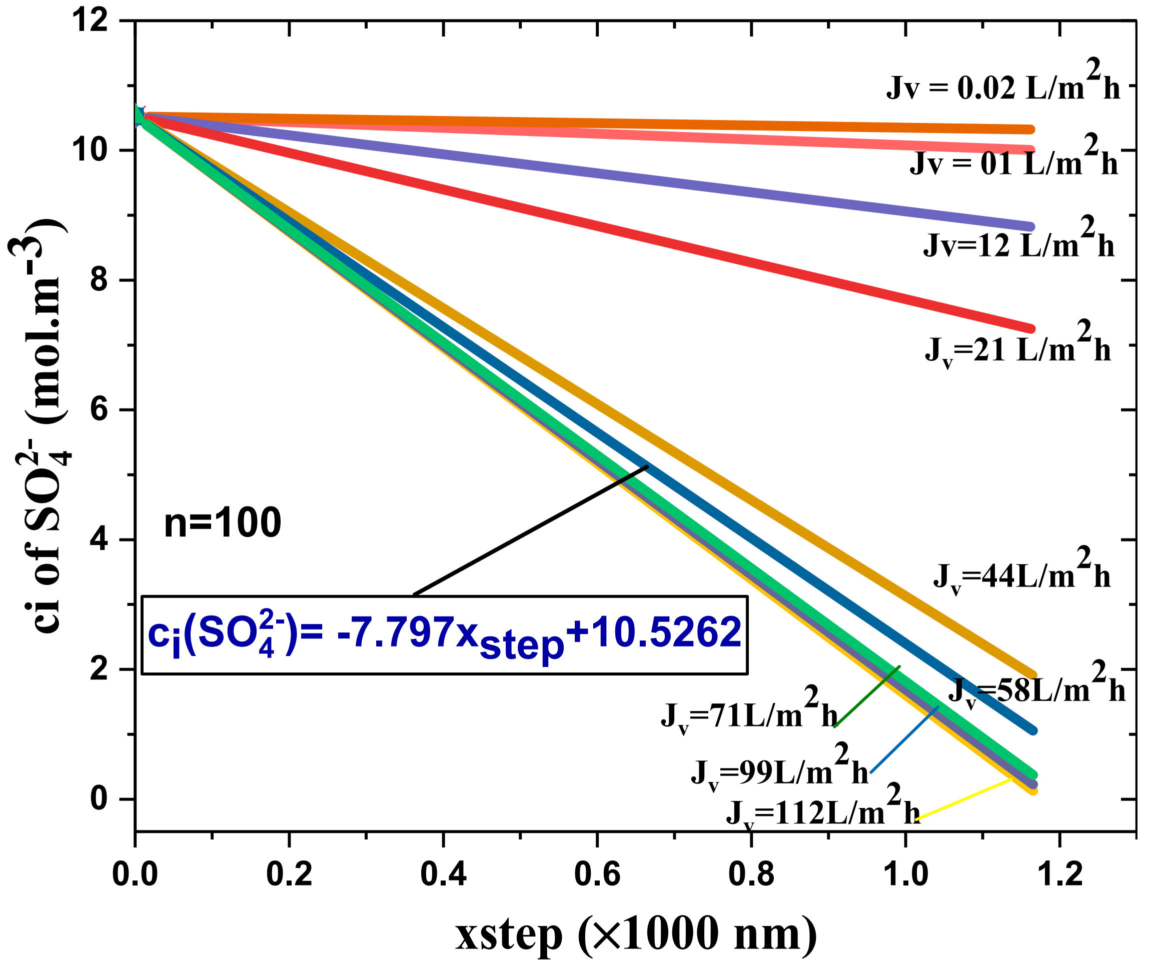

Figure S2: SO_4^{2-} ion concentration inside the membrane active layer versus the step-size (for different volumetric flux (J_v)) – Number of steps equals 100.

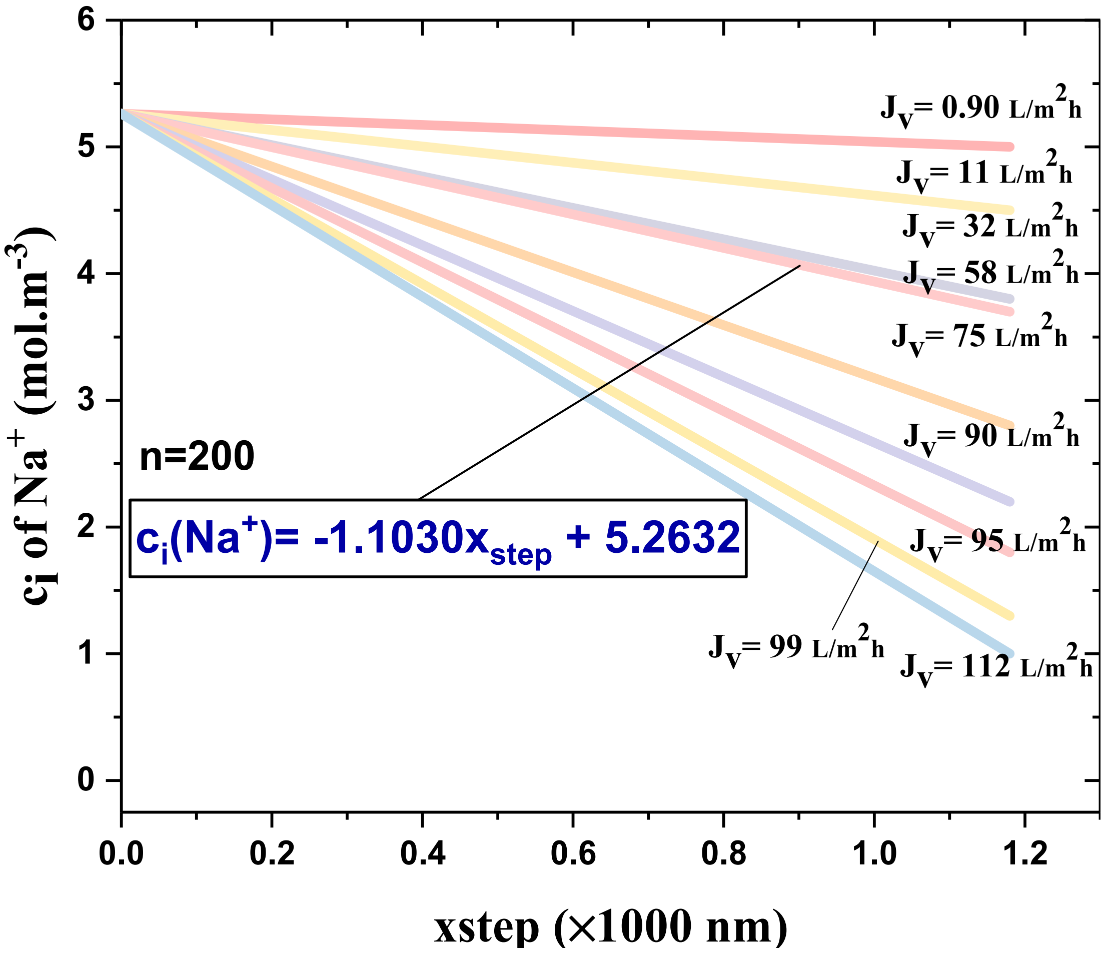

Figure S3: Na^+ ion concentration inside the membrane active layer versus the step-size (for different volumetric flux (J_v)) – Number of steps equal 200.

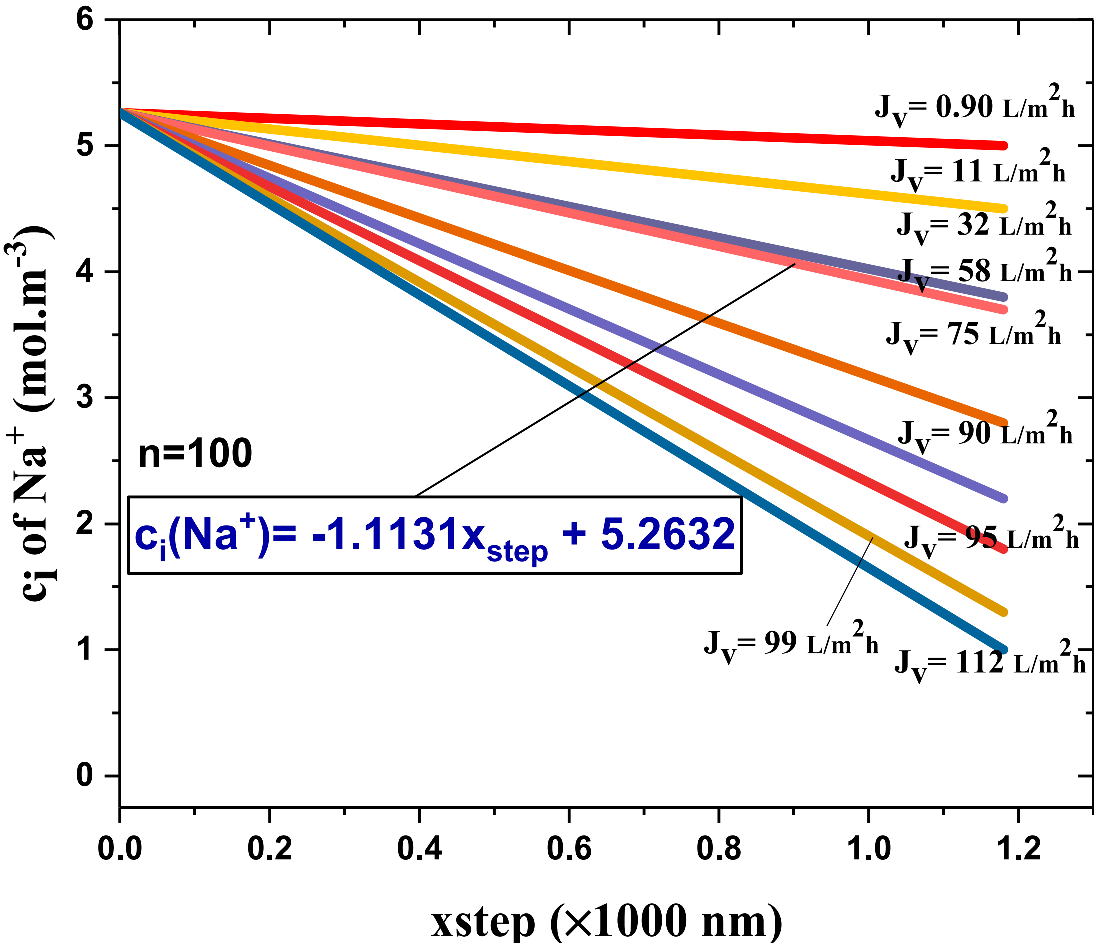

Figure S4: Na^+ ion concentration inside the membrane active layer versus the step-size (for different volumetric flux (J_v)) – Number of steps equal 100.
